# Supplementary material for: Mechanisms governing the pioneering and redistribution capabilities of the non-classical pioneer PU.1
Source: Nat Commun. 2020 Jan 21;11:402. doi: 10.1038/s41467-019-13960-2 (PMC6972792; doi:10.1038/s41467-019-13960-2)
Supplement: Supplementary file 7 — Source data [file 41467_2019_13960_MOESM7_ESM.zip › Source_Data/Figure5/Figure5A_MotifScanOutput/homerResults/motif40.similar.html]

motif40

## Information for motif40

T
A
G
C
G
T
A
C
G
A
T
C
G
T
A
C
G
A
T
C
T
G
A
C
G
A
T
C
G
T
A
C
G
T
A
C
  
Reverse Opposite:  

C
A
T
G
C
A
T
G
C
T
A
G
A
C
T
G
C
T
A
G
C
A
T
G
C
T
A
G
C
A
T
G
A
T
C
G
  

|  |  |
| --- | --- |
| p-value: | 1e-35 |
| log p-value: | -8.215e+01 |
| Information Content per bp: | 1.533 |
| Number of Target Sequences with motif | 1111.0 |
| Percentage of Target Sequences with motif | 36.84% |
| Number of Background Sequences with motif | 11884.3 |
| Percentage of Background Sequences with motif | 26.05% |
| Average Position of motif in Targets | 254.2 +/- 207.5bp |
| Average Position of motif in Background | 207.1 +/- 128.2bp |
| Strand Bias (log2 ratio + to - strand density) | -0.0 |
| Multiplicity (# of sites on avg that occur together) | 1.68 |
| Motif File: | file (matrix) reverse opposite |

### Similar de novo motifs found

|  |  |  |  |  |  |  |  |
| --- | --- | --- | --- | --- | --- | --- | --- |
| Rank | Match Score | Redundant Motif | P-value | log P-value | % of Targets | % of Background | Motif file |
| 1 | 0.931 | T A G C A G T C A G T C G A T C G T A C G A T C G T A C G T A C | 1e-34 | -80.056782 | 19.73% | 11.59% | motif file (matrix) |
| 2 | 0.835 | A C T G A C T G C A T G C A T G A C T G A C T G C T A G C T A G C T A G C T A G T A C G | 1e-30 | -70.622809 | 31.30% | 21.85% | motif file (matrix) |
| 3 | 0.777 | A G T C A G T C A G T C A G T C A G T C A G T C A G T C A G T C A G T C A G T C A G T C A G T C | 1e-20 | -48.308688 | 11.84% | 6.85% | motif file (matrix) |
